# Aqueous and alcoholic extracts of *Artemisia annua* L. improved insulin resistance via decreasing TNF-alpha, IL-6 and free fatty acids in high-fat diet/streptozotocin-induced diabetic mice 

**DOI:** 10.22038/AJP.2021.18829

**Published:** 2022

**Authors:** Mahshid Ghanbari, Forouzan Sadeghimahalli

**Affiliations:** 1 *Department of Toxicology, School of Pharmacy, Mazandaran University of Medical Sciences, Sari, Iran*; 2 *Department of Physiology, School of Medicine, Mazandaran University of Medical Sciences, Sari, Iran*; 3 *Immunogenetics Research Center, School of Medicine, Mazandaran University of Medical Sciences, Sari, Iran*; 4 *Cellular and Molecular Research Center, School of Medicine, Mazandaran University of Medical Sciences, Sari, Iran*

**Keywords:** Adipocytokins, Insulin sensitivity, Medicinal plant, Diabetes, Artemisia annua

## Abstract

**Objective:**

Type 2 diabetes mellitus (T2DM) is a metabolic disease that influences many people worldwide. Management of insulin resistance in T2DM without side effects of chemical drugs, is the ultimate goal of the medical community. *Artemisia annua* L. is used for the treatment of diabetes in folkloric medicine. The present study investigated the effects of aqueous and alcoholic extracts of *A. annua* (AA) on insulin resistance in high-fat diet/STZ-induced diabetic mice.

**Material and Methods:**

Mice were divided into groups including control with a normal diet, un-treated high-fat diet/streptozotocin-induced diabetic mice, and diabetic mice treated by oral administration of 100, 200, and 400 mg/kg body weight of water (hot and cold) and alcoholic extracts of AA. After four weeks of treatment with AA, blood sampling was carried out to measure factors involved in insulin resistance such as low-density lipoprotein/ High-density lipoprotein (LDL/HDL) ratio, free fatty acids, Tumor necrosis factor alpha (TNF-alpha), interleukin-6 (IL-6), and homeostasis model assessment of insulin resistance (HOMA-IR) as an index of insulin resistance.

**Results:**

The results showed that all AA extracts (100, 200, and 400 mg/kg) and metformin (250 mg/kg) significantly reduced the serum levels of free fatty acids, TNF-alpha, IL-6, LDL/HDL ratio, and HOMA-IR in diabetic mice compared to untreated diabetic mice (p<0.0001). Notably, the 400 mg/kg dose of cold-water extract was more effective than metformin in reduction of TNF-alpha and IL-6 (p<0.01 and p<0.05, respectively).

**Conclusion:**

These data illustrated that AA extracts attenuated insulin resistance by reducing the lipid profile and adipocytokines.

## Introduction

Insulin resistance (IR) is a major implication in the pathogenesis of type 2 diabetes mellitus (T2DM) (Sah et al., 2016 ▶; Zhao et al., 2018 ▶). IR produces unfavorable conditions in which insulin-responsive cells, including adipocytes and skeletal muscle, are not able to respond to the normal level of blood insulin (Yaribeygi et al., 2019 ▶). Different factors are involved in the development of IR, such as free fatty acid (FFA), low-density lipoproteins (LDL), and pro-inflammatory cytokines such as tumor necrosis factor-alpha (TNF-alpha) and interleukin-6 (IL-6) (Liu et al., 2016 ▶; Sah et al., 2016 ▶; Yaribeygi et al., 2019 ▶)

*Artemisia anuua* (AA) L. is a traditional Chinese medicinal herb. It is a species in the genus *Artemisia* that belongs to the Asteraceae family which includes over 500 species distributed throughout the world (Choi et al., 2013 ▶). It is also known as sweet wormwood, sweet annie, sweet sagewort and annual wormwood (in Chinese *qngho*). It is widely used as a dietary spice, herbal tea and medicinal plant in the mild climates of Asia, such as China and Korea (Das, 2012 ▶; Li et al., 2015 ▶; Kim et al., 2016 ▶; de Magalhães et al., 2012 ▶). Many studies reported that different species of *Artemisia*, which have been used in traditional medicine in East Asia, possess several bioactive functions such as antidiabetic (Nofal et al., 2009 ▶; Ghazanfar et al., 2014 ▶; Jung et al., 2007 ▶; Ogbonna et al., 2017 ▶; Kang et al., 2008 ▶), antihyperglycemic (Ribnicky et al., 2006 ▶; Issa and Hussen Bule, 2015 ▶) antihyperlipidemic (Vandanmagsar et al., 2014 ▶; Sah et al., 2016 ▶), and anti-insulin resistance activities (Vandanmagsar et al., 2014 ▶; Kheterpal et al., 2014 ▶; Richard et al., 2014 ▶). In addition, some studies have investigated the effect of AA extracts in diabetic animal models. In alloxan-induced diabetic rats, the aqueous extract of AA reduced fasting blood glucose, insulin, homeostasis model assessment-estimated insulin resistance (HOMA-IR), and LDL/HDL ratio as a predictor of coronary heart disease (Helal et al., 2014 ▶). Artemether, as one of the semi-synthetic artemisinin derivatives, improved insulin resistance and glucose homeostasis in type 2 diabetic db/db mice (Guo et al., 2018 ▶). Also, AA combined with the other plants' extracts reduced hyperglycemia in alloxan-induced diabetic rats (Ogbonna et al., 2017 ▶).

The hypothesis of the present study is that AA extracts reduce insulin resistance via decreasing lipid profile and/or adipocytokines in type 2 diabetic mice. So, the present study investigated the effect of hot-water, cold-water (Tonk et al., 2006 ▶), and alcoholic extracts of AA on risk factors developing insulin resistance. HOMA-IR as an index of insulin resistance and LDL/HDL ratio as a risk ratio, and serum concentrations of FFA, TNF-alpha, and IL-6 as factors involved in insulin resistance, were measured in high-fat diet/STZ-induced type 2 diabetic (HFD/STZ-induced T2D) mice.

## Materials and Methods

**Animals**

Male albino mice weighting 30-35 g (8-10 weeks of age) were housed under standard conditions (12 hr light/dark, at 22±2°C). Food and tap water *ad libitum* were available to the mice. The macronutrient contents of standard pellet mice diet (Beparvar production and distribution of animal feed company, Iran), consisted of carbohydrates (72.1%), proteins (22.1%), and lipids (7.5%) with a total caloric value of ~ 2900 kcal/kg. The composition of the HFD consisted of carbohydrates (27.5%), proteins (14.5%), and lipids (58.5%) with a total caloric value of ~ 4700 kcal/kg. All procedures performed in the present study were done in accordance with the local Ethics Committee of Mazandaran University of Medical Sciences, Sari, Iran (IR.MAZUMS.IMAMHOSPITAL.REC.1398.5758 and IR.MAZUMS.IMAMHOSPITAL.REC.1398.5761). 

Experimental animals were randomly allocated to 12 groups with five animals in each group, including: Control (CON): The animal were injected intraperitoneally (ip) with saline (0.9% w/w) used as an extracting solvent, once daily for four weeks; Type 2 diabetes (T2D): Diabetic animals injected (ip) with saline once daily for four weeks; T2D+Metformin (T2D+MTF): Diabetic animals treated with metformin (as a standard drug in treating T2D) 250 mg/kg (ip) once daily for four weeks; and diabetic groups treated with doses of 100, 200 and 400 mg/kg of hot-water (T2D+HWE), cold-water (T2D+CWE) and alcoholic (T2D+ALE) extracts of AA (orally, once a day for 4 weeks) (Kadi et al., 2019 ▶; Honmore et al., 2015 ▶).

**Plant material**

Aerial parts of AA were collected from the north of Iran in the months of May and June 2018. Genus and scientific species of plant were biotechnicaly identified by Dr. Masod Azadbakht Prof. of Sana University of Mazandaran, Sari, Iran and with record number: E1-39-2191 was preserved in the School of Pharmaceutics, Mazandaran University of Medical Sciences, Sari, Iran. Fresh aerial parts of AA were cleaned and dried at room temperature and then ground into powder using a grinding mill. Powdered aerial parts of AA were stored.

**Preparation of Aqueous and Alcoholic extracts of AA**

In this part, 450 ml of distilled water was added to 500 g obtained powder. Then, extraction was done away from sunlight during three days at room temperature. For providing hot-water extract, the mixture was boiled for 6 hr and, after that, filtered; then, the obtained extracts were filtered and concentrated using a rotary apparatus. The concentrated extract was frozen and dried. The dried extracts were stored in a glass container at -20°C.

Then, 400 ml of methanol 95% was added to 500 g dried-herb. Then extraction was done after 48 hr. The obtained extract was filtered and then concentrated by drying in a balloon using a rotary apparatus. The concentrated extract was frozen and dried. The dried extracts were stored in a glass container at -20°C.

**Induction of type 2 diabetes**

Diabetic groups of mice were fed with a high-fat diet (HFD) for eight weeks to induce hyperglycemia. After that, for producing sustained hyperglycemia, mice were injected with a single dose of streptozotocin (STZ) (65 mg/kg body weight, ip) (Sigma-Aldrich, USA). After ten days, blood glucose was measured by glucometer (On-Call EZ, USA). Mice with a blood glucose level ≥300 mg/dl, were considered stable hyperglycemic and used for this study (Gao et al., 2019 ▶; Zhang et al., 2011 ▶).

**Measurement of factors involved in insulin resistance**

After the end of the treatment period (4 weeks), following 16 hr overnight fasting, a deep anesthesia was induced anesthetized with xylazine/ketamine (10/110, mg/kg), blood sampling was done from the heart and the anesthetized mice were euthanized. The serum samples obtained by centrifuging (664×g) were separated and kept at -70°C to measure blood parameters, including HOMA-IR, LDL/HDL ratio, FFA, IL-6, TNF-alpha. The serum samples were analyzed for insulin, glucose, FFA, LDL, HDL, IL-6, and TNF-alpha concentrations by insulin Elisa kit (ZelBio, Germany), FFA Elisa kit (ZelBio, Germany), IL-6, and TNF-alpha Kit (ZelBio, Germany) and LDL and HDL kit (Pars Azmoon, Iran). The plasma glucose concentrations were determined using the glucose oxidase method (Pars Azmoon, Iran).

**HOMA-IR**

Homeostatic model assessment insulin resistance is one of insulin resistance indexes that are determined by a formula: HOMA-IR=(ci×cg)/22.5. Where ci is fasting insulin level (μU/ml) and cg is fasting glucose level (mmol/L) (Turner et al., 1979 ▶).

## Results

**The effect of metformin and AA extracts on serum levels of FA in HFD/STZ-induced diabetic mice**

**Figure 1:**
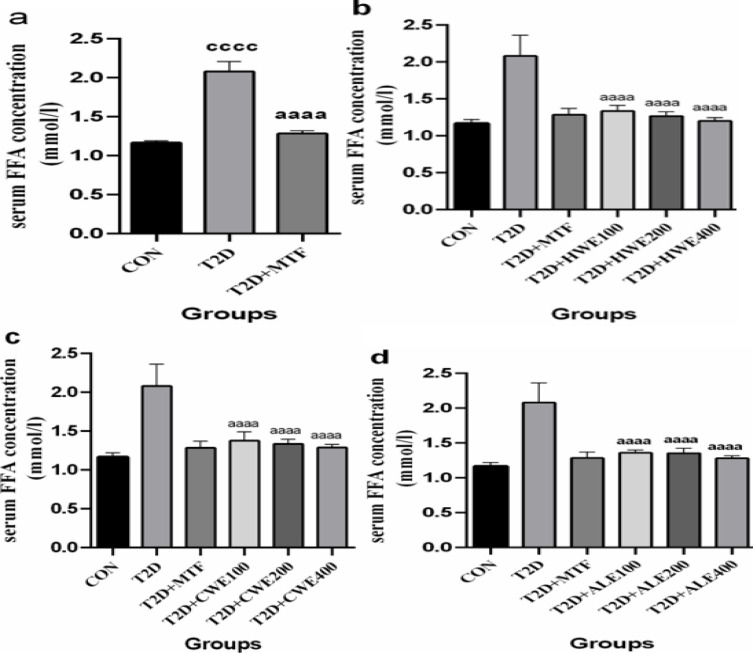
The effects of metformin (a) and various doses of hot-water (b), cold-water (c), and alcoholic (d) *Artemisia annua* extracts on the serum levels of free fatty acid (FFA) in high-fat diet/streptozotocin -induced diabetic mice. Data are expressed as the mean±SEM. (n=5). ^aaaa^P<0.0001 versus the T2D group. ^cccc^P<0.0001 versus the CON group. ^bbbb^P<0.0001 versus the T2D+MTF group. Groups: Control (CON), Type 2 diabetes (T2D), T2D+Metformin (T2D+MTF), T2D+hot-water extract (T2D+HWE), T2D+cold-water extract (T2D+CWE) and T2D+alcoholic extract (T2D+ALE)

Diabetic mice showed a marked increase in serum level of FA as compared to the control group (p<0.0001). These mice were treated with metformin (250 mg/kg) for four weeks. After treatment, serum concentrations of FFA decreased significantly in comparison with the T2D group (p<0.0001, Figure 1a). Doses of 100, 200, and 400 mg/kg of hot and cold-water and alcoholic extracts significantly reduced serum FFA concentration as compared to the T2D group (p<0.0001). The effect of all 3 doses of the extracts was the same, and there was no difference among them. Also, the effect of AA extracts was similar to metformin (Figure 1b, c, and d).

**The effect of metformin and AA extracts on serum levels of LDL/HDL ratio in HFD/STZ-induced diabetic mice**

**Figure 2:**
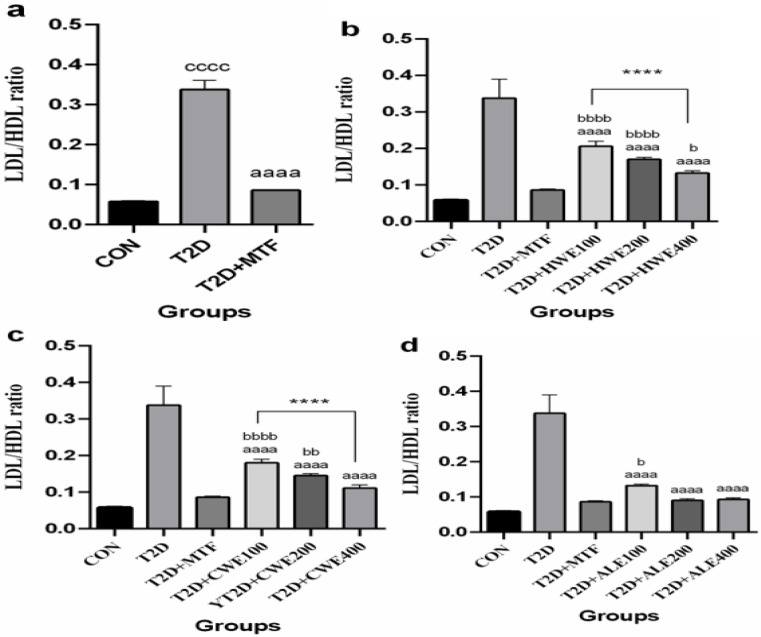
The effects of metformin (a) and various doses of hot-water (b), cold-water (c), and alcoholic (d) *Artemisia annua* extracts on the Low-density lipoprotein/ High-density lipoprotein (LDL/HDL) ratio in high-fat diet/streptozotocin -induced diabetic mice. Data are expressed as the mean±SEM. (n=5). ^aaaa^P<0.0001 versus the T2D group. ^cccc^P<0.0001 versus the CON group. ^b^P<0.05,^ bb^P<0.01, and ^bbbb^P<0.0001 versus the T2D+MTF group. ^****^P<0.0001 shows significant differences among doses of the same type of extract. Groups: Control (CON), Type 2 diabetes (T2D), T2D+Metformin (T2D+MTF), T2D+hot-water extract (T2D+HWE), T2D+cold-water extract (T2D+CWE) and T2D+alcoholic extract (T2D+ALE)

Induction of T2DM in mice increased LDL/HDL ratio significantly as compared to the control group (p<0.0001), but after treatment with metformin, this value significantly decreased (p<0.0001, Figure 2a).

Value of LDL/HDL following treatment with 100, 200, and 400 mg/kg of hot and cold-water and alcoholic extracts significantly decreased in comparison with the T2D group (p<0.0001) (Figure 2b, c, d). The impact of 400 mg/kg dose of hot-water extract was closer to that of metformin. This difference was slightly significant (p<0.05), while the difference at doses of 100 and 200 mg/kg was more significant (p<0.0001). On the other hand, the comparison of doses indicated that 400 mg/kg of hot-water extract was more effective than 100 mg/kg of hot-water extract in lowering the value of LDL/HDL (p<0.0001, Figure 2b).

There was a significant difference between doses of 200 mg/kg (p<0.01) and 100 (p<0.0001) of cold-water extract and the T2D+MTF group. Effect of 100 and 200 was the same, but dose 400 mg/kg was more effective than 100 (p<0.0001) in LDL/HDL ratio decline (Figure 2c).

The impacts of 400 and 200 mg/kg of alcoholic extract doses were similar to that of metformin; nevertheless, a significant difference was observed between the dose of 100 and the T2D+MTF group (p<0.05, Figure 2d). 

**The effect of metformin and AA extracts on serum levels of TNF-alpha in HFD/STZ-induced diabetic mice**

**Figure 3:**
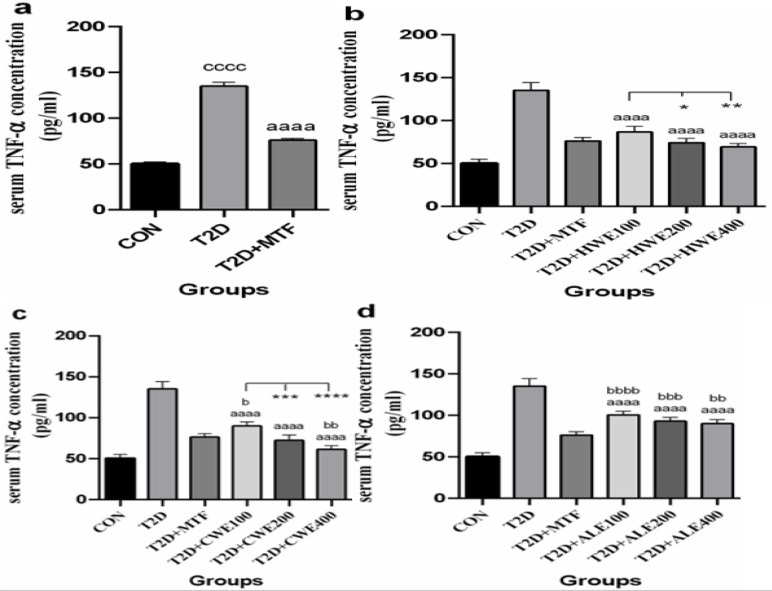
The effects of metformin (a) and various doses of hot-water (b), cold-water (c), and alcoholic (d) *Artemisia annua* extracts on the serum levels of Tumor necrosis factor alpha (TNF-alpha) in high-fat diet/streptozotocin-induced diabetic mice. Data are expressed as the mean±SEM. (n=5). ^aaaa^P<0.0001 versus the T2D group. ^cccc^P<0.0001 versus the CON group. ^b^P<0.05,^ bb^P<0.01, ^bbb^P<0.001, and ^bbbb^P<0.0001 versus the T2D+MTF group. ^*^P<0.05, ^**^P<0.01, ^***^P<0.001, and ^****^P<0.0001 show significant differences among doses of the same type of extract. Groups: Control (CON), Type 2 diabetes (T2D), T2D+Metformin (T2D+MTF), T2D+hot-water extract (T2D+HWE), T2D+cold-water extract (T2D+CWE) and T2D+alcoholic extract (T2D+ALE)

After induction of T2DM in mice, the serum concentration of TNF-alpha significantly increased as compared to the control group (p<0.0001) and after treatment with metformin, it was decreased (p<0.0001, Figure 3a).

All three treatment doses of hot and cold-water and alcoholic extracts in diabetic mice significantly reduced serum TNF-alpha in comparison with the T2D group (p<0.0001, Figure 3b, c, and d). Comparison of different doses revealed that 200 and 400 mg/kg of hot-water extract significantly decreased, TNF-alpha as compared to the 100 mg/kg dose (respectively, p<0.05 and p<0.01). However, between metformin and different doses of the hot-water extract, there was no significant difference (Figure 3b).

The impact of the 200 mg/kg dose of cold-water extract was similar to that of metformin. Notably, the dose of 100 mg/kg showed a weaker impact as compared to metformin (p<0.05) and the dose of 400 mg/kg was more effective than metformin in lowering TNF-alpha (p<0.01). Analysis of difference among groups showed that doses of 200 and 400 mg/kg reduced TNF-alpha value more than 100 mg/kg dose (respectively, p<0.001 and p<0.0001, Figure 3c).

A significant difference was observed between the T2D+MTF group and alcoholic extract doses of 100 mg/kg (p<0.0001), 200 (p<0.001), and 400 mg/kg (p<0.01). No significant difference was found among different doses of alcoholic extract. (Figure 3d). 

**The effect of metformin and AA extracts on serum levels of IL-6 in in HFD/STZ-induced diabetic mice**

**Figure 4:**
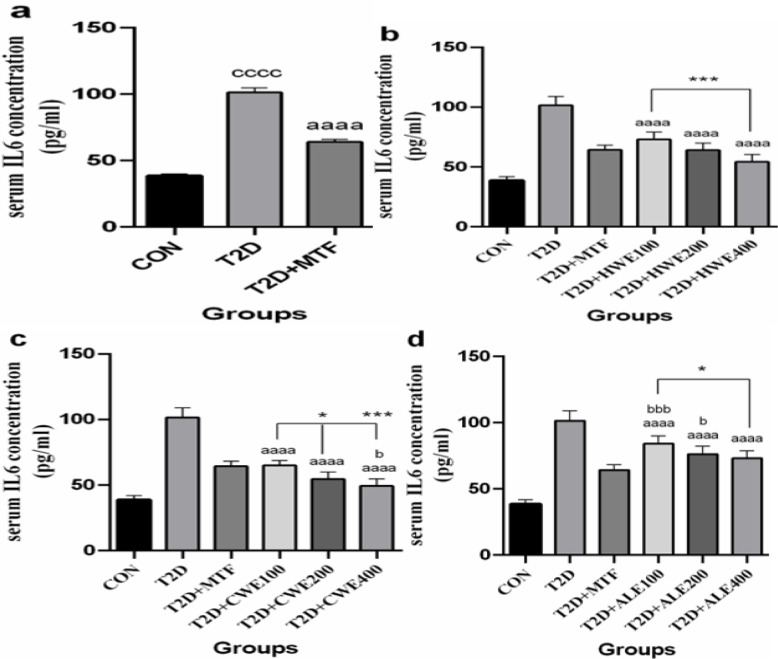
The effects of metformin (a) and various doses of hot-water (b), cold-water (c), and alcoholic (d) Artemisia annua extracts on the serum levels of interleukin-6 (IL-6) in high-fat diet/streptozotocin-induced diabetic mice. Data are expressed as the mean±SEM. (n=5). aaaaP<0.0001 versus the T2D group. ccccP<0.0001 versus the CON group. bP<0.05 and bbbP<0.001 versus the T2D+MTF group. *P<0.05 and ***P<0.001 show significant differences among different doses of the same type of extract. Groups: Control (CON), Type 2 diabetes (T2D), T2D+Metformin (T2D+MTF), T2D+hot-water extract (T2D+HWE), T2D+cold-water extract (T2D+CWE) and T2D+alcoholic extract (T2D+ALE)

After induction of T2DM in mice, the serum concentration of IL-6 significantly increased when compared to the control group (p<0.0001) but decreased after treatment with metformin (p<0.0001, Figure 4a).

The serum concentration of IL-6 significantly increased following the treatment with all 3 doses of hot and cold-water and alcoholic extracts in diabetic mice (p<0.0001, Figure 4b, c, and d).

No significant difference was observed between the doses of hot-water extracts and metformin. However, 400 mg/kg dose of hot-water extract with a more marked difference reduced IL-6 level when compared with 100 mg/kg dose of hot-water extract (p<0.001, Figure 4b).

The effect of doses of 100 and 200 mg/kg of cold-water extract was similar to the metformin. Nevertheless, effect of 400 mg/kg dose in reduction of IL-6 was more significant than metformin (p<0.05). Doses of 200 and 400 showed a more decreasing effect than the dose of 100 mg/kg (p<0.05 and p<0.001, respectively, Figure 4c).

The dose of 400 mg/kg of alcoholic extract had a similar lowering effect to metformin, while doses of 200 and 100 mg/kg showed a smaller lowering effect (p<0.05 and p<0.001, respectively, Figure 4d). 

**The effect of metformin and AA extracts on HOMA-IR in in HFD/STZ-induced diabetic mice**

**Figure 5:**
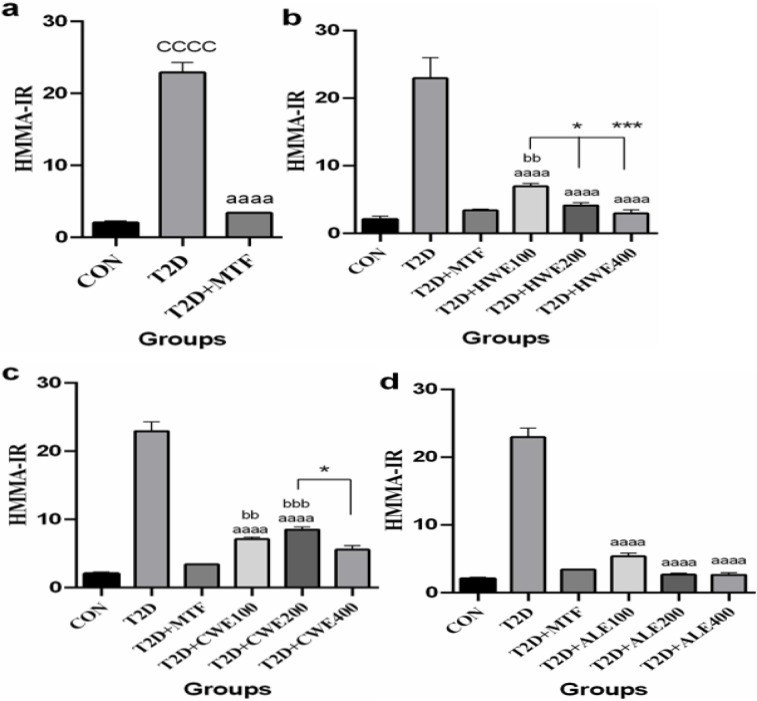
The effects of metformin (a) and various doses of hot-water (b), cold-water (c), and alcoholic (d) *Artemisia annua* extracts on homeostasis model assessment of insulin resistance (HOMA-IR) in high-fat diet/streptozotocin-induced diabetic mice. Data are expressed as the mean±SEM. (n=5). ^aaaa^P<0.0001 versus the T2D group. ^cccc^P<0.0001 versus the CON group. ^bb^P<0.01 and ^bbb^P<0.001 versus the T2D+MTF group. ^*^P<0.05, ^**^P<0.01 and ^***^P<0.001 show significant differences among different doses of the same type of extract. Groups: Control (CON), Type 2 diabetes (T2D), T2D+Metformin (T2D+MTF), T2D+hot-water extract (T2D+HWE), T2D+cold-water extract (T2D+CWE) and T2D+alcoholic extract (T2D+ALE)

HOMA-IR as an index of insulin resistance after induction of T2DM increased significantly when compared to the control group (p<0.0001); however, following treatment with metformin, it was decreased significantly (p<0.0001, Figure 5a).

ALL three doses of hot and cold-water and alcoholic extracts significantly reduced HOMA-IR in comparison with T2D group (p<0.0001). A significant difference was shown when 100 mg/kg of hot-water extract was compared to metformin, and doses of 200, and 400 mg/kg (respectively, p<0.01, p<0.05, and p<0.001, Figure 5b).

A significant difference was found between metformin and the doses of 100 and 200 mg/kg of cold-water extract (p<0.01 and p<0.001, respectively). Furthermore, the 400 mg/kg dose was more effective than 200 mg/kg (p<0.05, Figure 5c).

However, there was no significant difference between metformin, and 100, 200, and 400 mg/kg doses of alcoholic extract (Figure 5d). 

**Percent of changes induced by of metformin and AA extracts in HFD/STZ-induced diabetic mice**

The range of reduction (%) of FFA, LDL/HDL ratio, TNF-alpha, IL-6 levels, and HOMA-IR in mice treated by hot and cold-water, and alcoholic extracts, and metformin is presented in Table 1.

**Table 1:** Percent of HOMA-IR, LDL/HDL ratio, FFA, TNF-alpha, and IL-6 following treatment with metformin and *Artemisia annua* extracts in high-fat diet/streptozotocin-induced diabetic mice

Groups	Reduction FFA (%)	Reduction LDL/HDL (%)	Reduction TNF-alpha (%)	Reduction IL-6 (%)	Reduction HOMA-IR (%)
T2D+MTF	87.91	89.28	69.58	59.24	93.63
T2D+HWE100	82.41	46.43	71.12	45.22	66.75
T2D+HWE200	89.01	60.71	71.93	59.55	90.18
T2D+HWE1400	96.70	75.00	77.60	75.16	95.83
T2D+CWE100	76.92	57.14	53.30	57.96	75.90
T2D+CWE200	82.41	67.86	74.30	74.52	69.33
T2D+CWE400	86.81	82.14	87.26	83.12	83.33
T2D+ALE100	79.12	75.00	40.80	27.70	82.70
T2D+ALE200	80.22	89.28	49.76	40.13	95.44
T2D+ALE400	87.91	89.30	53.30	45.22	97.27

T2D: type 2 diabetes; T2D+MTF: T2D+Metformin; T2D+HWE: T2D+hot-water extract; T2D+CWE: T2D+cold-water extract; T2D+ALE: T2D+alcoholic extract; HOMA-IR: assessment of insulin resistance; LDL/HDL: low-density lipoprotein/high-density lipoprotein ratio; FFA: free fatty acid; TNF-alpha: tumor necrosis factor alpha; IL-6: interleukin-6

## Discussion

In the present study, we found that induction of T2D using high-fat diet and injection of STZ in mice produced insulin resistance. It increased the factors involved in insulin resistance, including blood levels of FFA, LDL/HDL ratio, TNF-alpha, IL-6, and HOMA-IR; however, these changes were reversed by metformin and water and alcoholic extracts of AA. 

Our results indicated that the index of insulin resistance, HOMA-IR, was remarkably decreased in HFD/STZ-induced diabetic mice after treatment with MTF and AA. The efficacy of the extracts' doses was similar to metformin in reducing HOMA-IR. Several studies indicated that AA extracts or their bioactive compounds lowered HOMA-IR. For example, myricetin, as one of the flavonoids obtained from AA extract, improved the insulin sensitivity in mice and rats fed with HFD (Liu et al., 2007 ▶; Choi et al., 2014 ▶; Kim et al., 2016 ▶). Helal et al have shown that aqueous extract of AA in alloxan-induced diabetic rats decreased HOMA-IR (Helal et al., 2014 ▶). In a human study, 10 weeks of treatment with *Artemisia scoparia* extract in the form of a tablet which contained 200 mg of the extract (two tablets/day), reduced HOMA-IR and improved insulin sensitivity (Sun et al., 2016 ▶) in women with gestational diabetes. In T2D mice induced by HFD, high levels of HOMA-IR were reversed following chronic oral treatment with *Artemisia herba-alba* Asso given for 18 weeks (Hamza et al., 2011 ▶).

In the present study, all extracts of AA showed an antilipidemic effect in terms of a notable decrease in the LDL/HDL ratio and serum level of FFA in HFD/STZ-induced diabetic mice. Antilipidemic efficacy of most doses of the extracts was similar to metformin. 

In T2DM conditions, because of impaired insulin action, lipolysis increases in adipose tissue paralleled with a decline of the activity of insulin-depended lipoprotein lipase, which leads to a high level of FFA in blood (Albasher et al., 2020 ▶). FFAs can directly interrupt insulin signaling pathways through stimulating protein kinase C isoforms, which in turn, impair the cellular mechanism of insulin action and finally inhibit the entrance of glucose into peripheral cells (Albasher et al., 2020 ▶; Kavitha et al., 2016 ▶; Yaribeygi et al., 2019 ▶). It seems that defects in insulin-induced glucose transport in skeletal muscles might be responsible for the induction of insulin resistance (Goldstein, 2002 ▶). Albasher et al. showed that HFD/STZ-induced diabetic rats treated with ethanolic extract of *Artemisia judaica* had reduced HDL but enhanced LDL levels in serum (Albasher et al., 2020 ▶; Ahmad et al., 2014 ▶; Sefi et al., 2010 ▶). According to previous studies, *Artemisia* species displayed a notable hypolipidemic activity, which was indicative of an improvement in insulin function (Kim et al., 2016 ▶). Furthermore, antihyperlipidemic activity was observed in HFD-induced diabetic mice treated with *Artemisia herba-alba* Asso (Hamza et al., 2011 ▶). Besides, oral treatment with 200 and 400 mg/kg doses of methanolic extract of *Artemisia indica* for 15 days in STZ-induced diabetic rats, reduced lipid profile (LDL) (Ahmad et al., 2014 ▶). Also, AA extract reduced the LDL/HDL ratio in alloxan-induced diabetic rats treated intragastrically with aqueous extract of AA, 2 times/day for 30 days (Helal et al., 2014 ▶). The hypolipidemic effect of AA in this study may be due to the bioactive components in extracts, such as flavonoids and polyphenols (Kim et al., 2016 ▶).

The present study results showed that adipocytokines, TNF-alpha, and IL-6 significantly decreased in HFD/STZ-induced diabetic group following treatment with metformin and AA. However, the treatment efficacy of cold-water extracts at the dose of 400 mg/kg, in reduction of TNF-alpha and IL-6, was remarkably higher than that of metformin. 

It is well known that the adipocytes synthesize and secret chemical messengers called ‘adipocytokines’, such as TNF-alpha and IL-6. Adipocytokines have been linked to insulin resistance associated with T2DM. TNF-alpha, both directly and indirectly via increasing the secretion of FFA, impairs insulin signaling (Yaribeygi et al., 2019 ▶; Goldstein, 2002 ▶). IL-6 in healthy individuals is a useful regulator of glucose metabolism. Whereas in obesity-associated to T2DM, IL-6 as an inflammatory factor probably increases the existing inflammation. So, it is etiologically involved in the pathogenesis of T2DM by impairing insulin signaling and β-cell function (Liu et al., 2016 ▶).

In agreement with the results of this study, *Artemisia dracunculus* L. extract ameliorated insulin sensitivity through attenuating pro-inflammatory cytokines in human skeletal muscle culture (Vandanmagsar et al., 2014 ▶). Also, this plant decreased the production of IL-6 in macrophages (Aggarwal et al., 2015 ▶). Another study showed that following treatment of HFD-STZ-induced diabetic rats with *Artemisia judaica* extract for 28 days, the level of pro-inflammatory cytokines such as TNF-alpha was reduced (Albasher et al., 2020 ▶). The lowering effect of AA extracts on adipocytokines may be due to the presence of flavonoids (Li et al., 2015 ▶). AA is one of the medical plants that consists of a high level of flavonoids. Flavonoid compounds have several actions such as antidiabetic effect, improving insulin sensitivity and increasing insulin release from β-cells (Kartikadewi et al., 2019 ▶; Nathan et al., 2007 ▶; Albasher et al., 2020 ▶, Guo et al., 2018 ▶).

It has been proven that medical plants such as AA, because of having the bioactive compounds, particularly flavonoids, decrease the complications of diabetes mellitus, especially T2D (Das, 2012 ▶). All effects mentioned in the present study may be attributed to flavonoids. One of the flavonoids present in the extract of AA is myricetin, which has antioxidant and insulin-sensitivity improving properties (Kim et al., 2016 ▶). Two other flavonoids present in the AA extract are casticin and chrysosplenol D (Li et al., 2015 ▶); furthermore, anti-inflammatory compounds in AA reduce the production of pro-inflammatory cytokines (de Magalhães et al., 2012 ▶). It can be suggested that the AA extract thanks to it bioactive components, via reduction of lipid profile, TNF-alpha, and IL-6, normalized insulin resistance and improved insulin sensitivity in HFD-STZ-induced diabetic mice.

In conclusion, all types of AA extracts (i.e. hot and cold-water and alcoholic), improved insulin resistance through reduction of LDL/HDL ratio, free fatty acids, TNF-alpha, and IL-6. Although the effectiveness of treatment with most doses of the extracts, especially higher doses, was similar to that of metformin, the efficacy of 400 mg/kg dose of cold-water extract was higher than metformin in reduction of TNF-alpha, and IL-6. These findings confirm that higher levels of bioactive components might be present in higher concentrations of these extracts. 

## Conflicts of interest

The authors have declared that there is no conflict of interest.
